# Expression Characterization of *AtPDI11* and Functional Analysis of AtPDI11 D Domain in Oxidative Protein Folding

**DOI:** 10.3390/ijms23031409

**Published:** 2022-01-26

**Authors:** Fenggui Fan, Hao Zhang, Qian Wei, Yahui Wei

**Affiliations:** 1 Key Laboratory of Resource Biology and Biotechnology in Western China, Ministry of Education & College of Life Sciences, Northwest University, Xi’an 710069, China; 18737310533@163.com; 2 Shaanxi Engineering Research Centre for Conservation and Utilization of Botanical Resources, Xi’an Botanical Garden of Shaanxi Province, Institute of Botany of Shaanxi Province, Xi’an 710061, China; weiqian@xab.ac.cn

**Keywords:** protein disulfide isomerase, AtPDI11, D domain, oxidative protein folding

## Abstract

The formation and isomerization of disulfide bonds mediated by protein disulfide isomerase (PDI) in the endoplasmic reticulum (ER) is of fundamental importance in eukaryotes. Canonical PDI structure comprises four domains with the order of **a**-**b**-**b′**-**a′**. In *Arabidopsis thaliana*, the PDI-S subgroup contains only one member, AtPDI11, with an **a**-**a′**-**D** organization, which has no orthologs in mammals or yeast. However, the expression pattern of AtPDI11 and the functioning mechanism of AtPDI11 **D** domain are currently unclear. In this work, we found that PDI-S is evolutionarily conserved between land plants and algal organisms. *AtPDI11* is expressed in various tissues and its induction by ER stress is disrupted in *bzip28/60* and *ire1a/b* mutants that are null mutants of key components in the unfolded protein response (UPR) signal transduction pathway, suggesting that the induction of *AtPDI11* by ER stress is mediated by the UPR signaling pathway. Furthermore, enzymatic activity assays and genetic evidence showed that the **D** domain is crucially important for the activities of AtPDI11. Overall, this work will help to further understand the working mechanism of AtPDI11 in catalyzing disulfide formation in plants.

## 1. Introduction

Most newly synthesized secretory and membrane proteins are folded in the endoplasmic reticulum (ER), in which an oxidative environment facilitates the formation of intramolecular disulfide bonds [1]. In eukaryotes, ER oxidoreductin-1 (Ero1) and protein disulfide isomerase (PDI) constitute a major oxidative protein folding pathway, in which Ero1s supply oxidizing equivalent from molecular oxygen to the active site of PDI, and oxidized PDI further catalyzes the formation of disulfide bonds in nascent proteins to ensure that only correctly folded proteins exit the ER [2,3].

Once the demand for protein folding exceeds the capacity of the ER, especially under adverse environmental conditions, the unfold protein response (UPR) signaling pathways will be activated [4,5,6]. In response to ER stress, basic leucine zipper domain (bZIP) transcription factor bZIP60 mRNA is spliced by INOSITOLREQUIRING PROTEIN 1 (IRE1), the major ER stress sensor, resulting in its migration to the nucleus [6]; in addition, bZIP28 is mobilized from the ER to Golgi where it is proteolytically cleaved by SITE-2-PROTEASE (S2P). The cleaved transcription factors bZIP60 and bZIP28 enter the nucleus to upregulate the expression of UPR related genes, such as *binding protein* (*BIP*), *calnexin* (*CNX*), and *PDI*, so as to facilitate protein folding and alleviate ER stress [7,8].

PDI is a multifunctional enzyme as it can catalyze free-thiol oxidation reaction and reduction or isomerization of disulfide bonds depending on their redox states [9]. Canonical PDI (EC5.3.4.1) is composed of four thioredoxin-like domains sharing the arrangement of **a**-**b**-**b′**-**a′**, with two **a**-type thioredoxin domains containing an active site motif -CGHC- and two **b**-type thioredoxin domains lacking an active site motif [10,11,12]. In Arabidopsis, there are 14 members in PDI family, and they have been classified into six structurally distinct subfamilies (A, B, C, L, M, and S) based on the number and position of the thioredoxin domains [12,13,14]. Six isoforms of PDI-L subgroups (named from AtPDI1to AtPDI6) share the **a**-**b**-**b′**-**a′** domain organization of canonical PDI [14]. Emerging evidence supports that PDI-L members are involved in plant responses to abiotic stress, such as drought, high salinity, high light, and so on [15,16,17,18]. AtPDI5 is also involved in embryo development by inhibiting cysteine proteases programmed cell death [19]. It is noteworthy that AtPDI2 is located in the nucleus and interacts with the nuclear transcription factor MEE8 (maternal effect embryo arrest 8), suggesting its function in embryo development [20]. PDI-M subgroup has two members, AtPDI9 and AtPDI10 with **a^0^**-**a**-**b** domain arrangement, which is required for pollen viability and normal exine formation in plants subjected to heat stress [21]. Although the expression patterns and enzyme activities of PDI-B (the sole member AtPDI8) and PDI-C subgroup (AtPDI7, AtPDI12, AtPDI13) have been identified, their physiological functions are still unclear [13,22,23]. Recently, using deletion mutation and biochemical analysis, we found that AtERO1 can interact with multiple AtPDIs, and AtPDI-L members mainly serve as an isomerase, while AtPDI-M/S members are more efficient in accepting oxidizing equivalents from AtERO1 and catalyzing disulfide bond formation. AtPDI-L and AtPDI-M/S subgroups work synergistically in catalyzing oxidative protein folding [24,25]. A similar model of cooperation of GmPDIM and GmPDIL-2 was also found in soybeans [26].

PDI-S is a unique plant subgroup with **a**-**a′**-**D** domain arrangement [14]. The **D** domain is analogous to the C-terminal domain of human ERp29 which has a conserved 5-α-helix hydrophobic region serving as a protein binding site [27]. In Arabidopsis, only one member of PDI-S subgroup, *AtPDI11*, was significantly upregulated by ER stresses [14]. The expression of truncated versions of AtPDI11 impedes embryo sac development, however, the null mutations of the AtPDI11 have no similar phenotype so the actual role of AtPDI11 in these processes remains unclear [28]. GmPDIS-1, the orthologs of AtPDI11 in soybean, is involved in the folding of proglycinin in seeds [29]. Recently, we proved that AtPDI11 is present in four redox states using a transient expression system, which is determined by the active site cysteines in the **a** and **a′** domains [30]. The **D** domain of AtPDI11 interacts with calreticulin 1 (CRT1) and calreticulin 2 (CRT2) and probably assists with the folding of glycoproteins in the ER [31]. Among the multiple PDI single mutants tested (*pdi1*, *pdi2*, *pdi5*, *pdi6*, *pdi9*, *pdi10*, and *pdi11*), only *pdi11* single mutants displayed obviously growth inhibition under reducing treatment [25]. All of the above implies that AtPDI11 is unique and plays an important role in plant oxidative protein folding. However, the regulation of *AtPDI11* expression remains unclear and its working mechanism is not fully understood.

In this work, we reported the evolutionary relationships of the PDI-S subgroup across Algae, Mosses, Gymnosperms, and Angiosperms. Tissue-specific expression of *AtPDI11* transcripts and its induction under ER stress were analyzed, and the function of **D** domain of AtPDI11 was studied. Our work provides a basis for further investigation on AtPDI11 function.

## 2. Results

### 2.1. Sequence Analysis of AtPDI11 and AtPDI11 Homologs from Various Species

**Figure 1:**
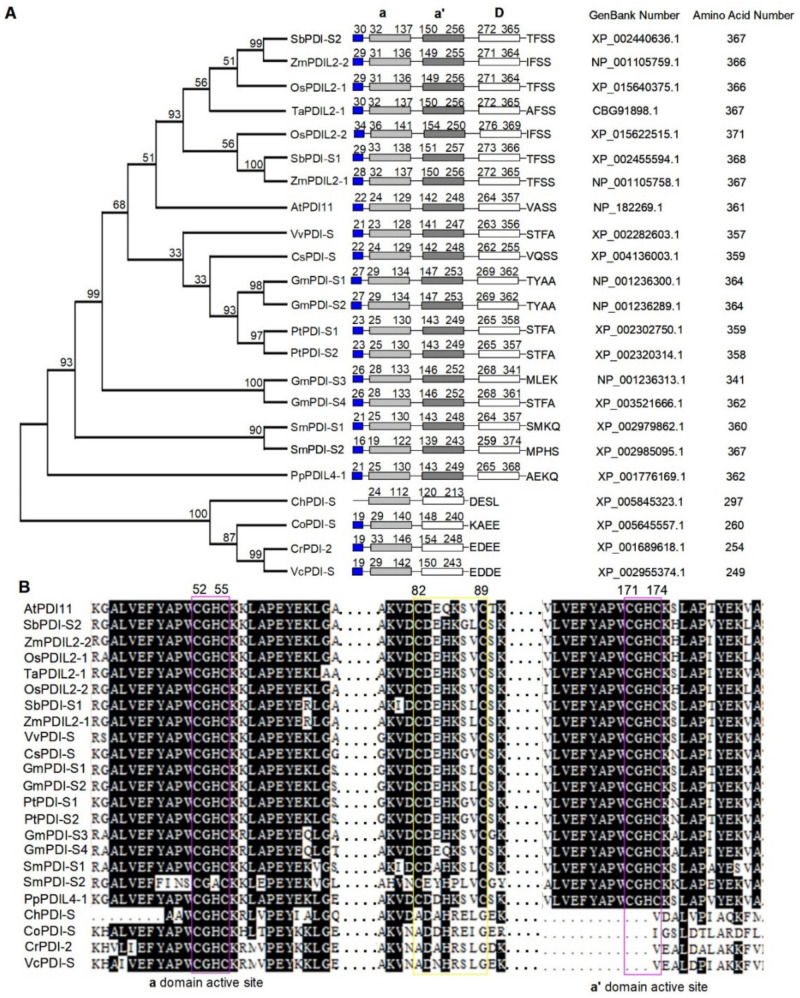
Phylogenetic analysis and protein sequence alignment of PDI-S from various species. (**A**) Phylogenetic analysis of AtPDI11 and its homologs in different species. A neighbor-joining phylogenetic tree was constructed using MEGA 7.0 software based on protein sequences of various AtPDI11 homologs. The numbers at each tree root are bootstrap values. At, *Arabidopsis thaliana*; Ch, *Chlorella variabilis*; Co, *Coccomyxa subellipsoidea*; Cr, *Chlamydomonas reinhardtii*; Cs, *Cucumis sativus*; Gm, *Glycine max*; Os, *Oryza sativa*; Pp, *Physcomitrella patens*; Pt, *Populus trichocarpa*; Sb, *Sorghum bicolor*; Sm, *Selaginella moellendorffii*; Ta, *Triticum aestivum*; Vv, *Vitis vinifera*; Vc, *Volvox carteri f. nagariensis*; Zm, *Zea mays*. (**B**) Multiple sequence alignment of AtPDI11 regions containing the active sites (lilac boxes) and the intermolecular disulfide bond Cys82_Cys89 (yellow box) from various species using the Clustal W program.

To investigate the evolutionary relationship of the PDI-S subgroup, altogether 23 PDI-S subgroup members from various species were compiled into a data matrix and aligned using MEGA7 software [32]. The phylogenetic tree derived from analysis of the matrix using the Poisson correction method is shown in Figure 1A. PDI-S subgroup has representatives with high similarity in all organisms going back to the algal. The cladogram is rooted with the PDI-S of the algal, including *Chlorella variabilis*, *Coccomyxa subellipsoidea*, *Chlamydomonas reinhardtii*, and *Volvox carteri f. nagariensis.* Such phylogenetic analyses are not surprising given the most ancient ancestor in the analysis. In addition, land plants possess an arrangement with **a**-**a′**-**D**, and algal organisms share an **a**-**D** domain arrangement, which is probably due to duplication of single domains during the evolutionary process. In sessile organism-formed clades, the moss and Fern AtPDI11 homolog (*Selaginella moellendorffii*) also clade separately in an outgroup on their own branch. After these outgroups at the base of the tree, the angiosperms branch off into their separate clades of monocotyledons (such as wheat, rice, maize, and sorghum) and dicotyledons (Grape, cucumber, soybean, and poplar), clearly separated from each other. Interestingly, dicotyledon *Arabidopsis thaliana* falls into the monocotyledon clade. Furthermore, amino acid sequence alignment showed that the active sites in two catalytic Trx domains are very well conserved in sessile organisms. We also found a conserved CysX_6_Cys motif between two type-**a** domains in various species except algae (Cys82X_6_Cys89 in Arabidopsis, X represents any amino acid) (Figure 1B). In Arabidopsis, the replacement of Cys by Ala in AtPDI11 Cys82X_6_Cys89 showed similar redox forms to wild-type protein, indicating that CysX_6_Cys motif forms a stable structural disulfide [30].

### 2.2. Histochemical Analysis Reveals Tissue-Specific Expression of AtPDI11 Transcripts

**Figure 2:**
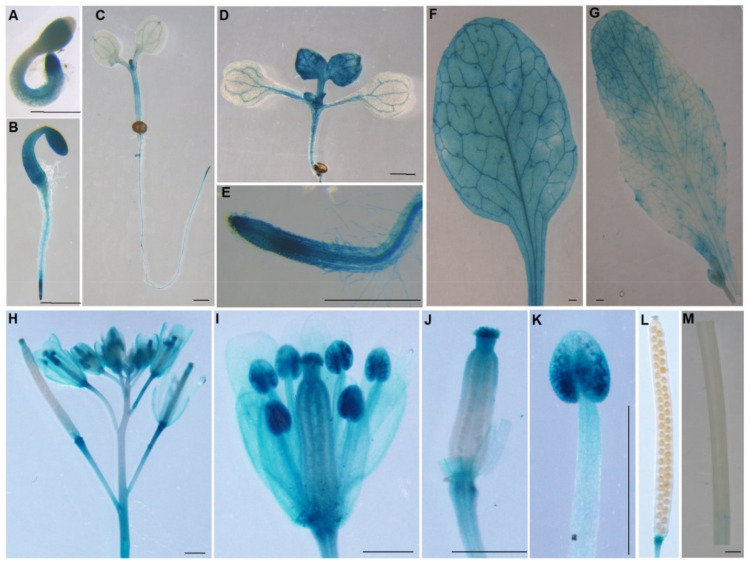
Spatial and temporal expression of AtPDI11. Spatiotemporal expression patterns of the AtPDI11 gene driven by its native promoter fused with GUS gene in transgenic plants. Promoter activity was visualized by histochemical GUS staining. (**A**) One-day-old seedlings. (**B**) Two-day-old seedlings. (**C**) Five-day-old seedlings. (**D**) Two-week-old seedling leaves. (**E**) Two-week-old seedling roots. (**F**) Rosette leaf of a four-week-old plant. (**G**) Cauline leaf of a four-week-old plant. (**H**–**L**) Inflorescence, flowers, stigma, anther, and siliques of five-week-old plant. (**M**) Stem of 5-week-old plant. Bar = 1 mm.

To analyze the expression patterns of *AtPDI11*, a transgenic plant with *β-glucuronidase* (*GUS*) gene driven by the promoter of *AtPDI11* (*ProPDI11*, 2.0 kb upstream of the start codon) was generated. As shown in Figure 2, The *ProPDI11* was active in all tissues of one- or two-day-old plants, especially in cotyledons, hypocotyls, and root apex (Figure 2A,B). In seedlings, the *ProPDI11* was weakly active in cotyledons compared with strongly expression in the root apex, apical meristems (in five-day-old-plant), and true leaf (two-week-old plant) (Figure 2C–E). *AtPDI11* is expressed in rosette and cauline leaves of four-week-old plants (Figure 2F,G). It has been shown that truncated AtPDI11 impedes embryo sac development [28], so we examined the expression of *ProPDI1::GUS* at inflorescences with flowers at progressive stages. As shown in Figure 2H–J, the *ProPDI11* was expressed in anthers, stigmas, sepals, and petals. In particular, GUS activity was much higher in anther due to the strong expression of *AtPDI11* in pollen (Figure 2K). Meanwhile, the *ProPDI11* was not active in silique or stem, but it was strongly active in abscission scars of sepals, petals, and stamens (Figure 2L,M). Taken together, *AtPDI11* is expressed in various tissues and at different development stages of plants.

### 2.3. Expression of AtPDI11 Is Induced by ER Stress

**Figure 3:**
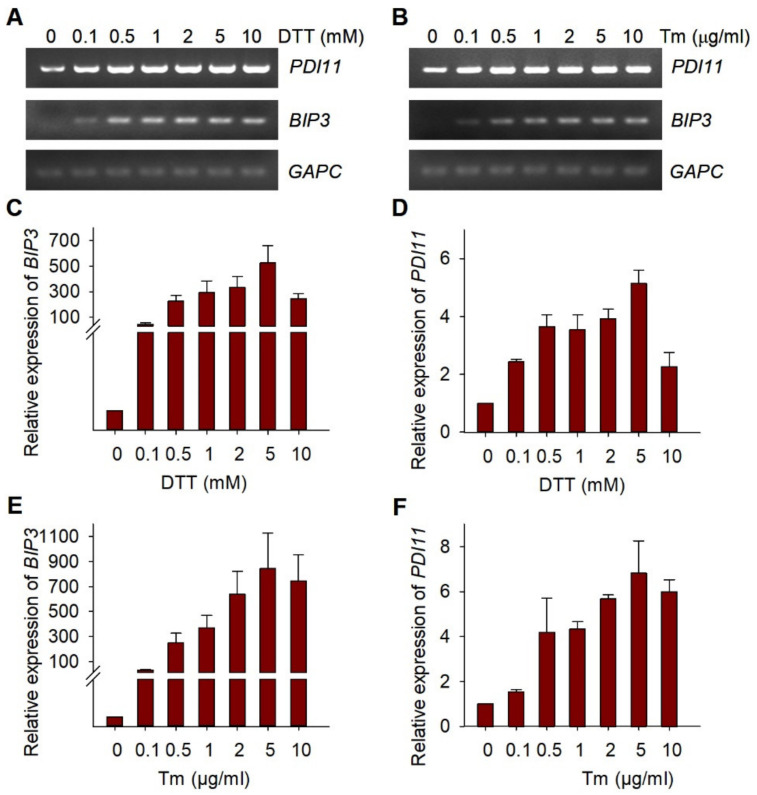
Expression level of *AtPDI11* under DTT/Tm treatment of the different concentration. (**A**,**B**) RT–PCR analysis of *AtPDI11* expression using 7-day-old wild-type plants under DTT (**A**) or Tm (**B**) treatment of different concentration for 5 h. A *GAPC* gene was used as a loading control. (**C–F**) Quantitative real-time PCR analysis of *AtPDI11* expression using 7-day-old wild-type plants under different concentration of DTT (**C**,**D**) or Tm (**E**,**F**) treatment for 5 h. The expression level of *AtBIP3* was used as a positive control. Data are shown as means ± SE from three independent biology repeats. The expression levels of *AtPDI11* and *AtBIP3* were normalized to expression level of *AtGAPC*.

The accumulation of unfolded or misfolded proteins in the ER can lead to ER stress that initiates UPR [8]. PDI can catalyze the formation of disulfide bonds and help proteins fold correctly. The transcription of several *AtPDI* genes was induced by UPR [14]. Then, we wanted to know how the expression of *AtPDI11* is changed when plants suffer from different degrees of ER stress. As shown in Figure 3A,B, as expected, abundance of *AtBIP3* transcripts measured by reverse-transcription PCR (RT–PCR) significantly increased under ER stress inducers Dithiothreitol (DTT) or Tunicamycin (Tm) treatment. Similarly, the transcript level of *AtPDI11* upon activation of the UPR was markedly higher than control. In order to more intuitively show the expression trend of *AtPDI11* under different DTT/Tm concentration stress, we conducted a more thorough quantitative RT-PCR (qRT–PCR) analyses of *AtPDI11* expression. Similar with RT-PCR, the expression level of *AtBIP3* and *AtPDI11* gradually increased under DTT or Tm treatment in a dose-dependent manner. It should be noted that the expression of *AtPDI11* under 10 mM DTT treatment decreased to the level observed under 0.1 mM DTT, and the *AtBIP3* transcript level also decline to some extent. This could be because ER stress caused by DTT treatment exceeded the tolerance of plants (Figure 3C–F).

**Figure 4:**
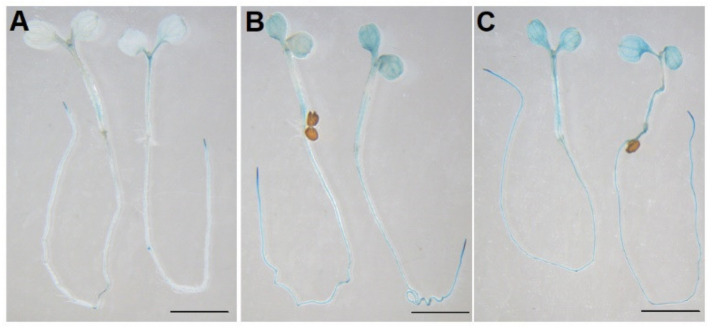
Comparison of GUS activity for *ProPDI11::GUS* transgenic plants under DTT or Tm treatment. Five-day-old seedlings of *ProPDI11::GUS* transgenic plants were treated with 2 mM DTT (**B**) or 5 μg/mL Tm (**C**) for 5 h and then immersed fully into the GUS staining solution for 1 h in dark. Seedlings without treatment were used as control (**A**). Bar = 2 mm.

We further tested the promoter activity of *AtPDI11* under ER stress. As shown in Figure 4, transgenic plants with the *AtPDI11* promoter fused to GUS reporter gene showed stronger GUS staining after being treated with 2 mM DTT and 5 μg/mL Tm than control conditions (Figure 4). This result indicates that significantly increased GUS activity was tested after DTT or Tm treatment. Hence, the induction of *AtPDI11* under ER stress is due to the activation of the *AtPDI11* promoter by UPR. Of course, we cannot rule out other factors affecting the increased expression of *AtPDI11* under ER stress.

Previously, we reported the growth inhibition of the *pdi11* mutant under DTT treatment [25,30]. As shown in Figure S1, with the increase of DTT concentration, both *pdi11* mutant lines (*pdi11-1* and *pdi11-2*) were dramatically inhibited compared to wild-type, especially in 2.0 mM DTT (Figure S1A,B). When exposed to Tm treatment, the growth of *pdi11* mutant lines and transgenic complementation lines (Com) were significantly inhibited in 50 ng/mL or 75 mg/mL, and positive control *bzip28/60* was more sensitive to Tm treatment compared with wild-type Col-0. It is worth noting that the extent of both *pdi11-1* and *pdi11-2* growth inhibition was similar to that of Col-0 (Figure S1C,D), although the expression of *AtPDI11* was significantly induced by Tm treatment (Figure 2B,F). These results further support our conclusion that AtPDI11 is more efficient in catalyzing disulfide bond formation and relatively weaker in catalyzing isomerization of the non-native disulfide bonds formed on the misfolded proteins [25].

### 2.4. The Induction of AtPDI11 by ER Stress Is Governed by Key UPR Signaling Mediators

**Figure 5:**
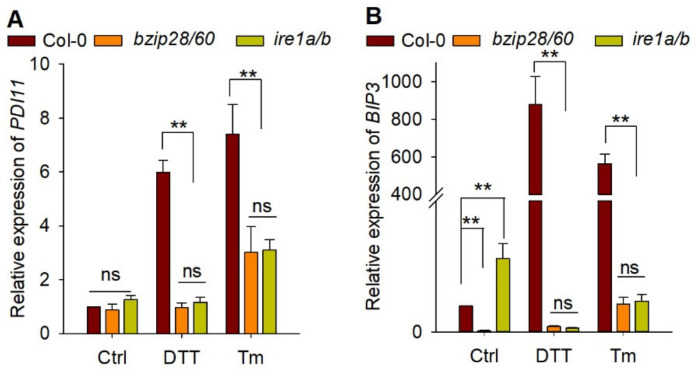
Quantitative real-time PCR analysis of *AtPDI11* expression in wild-type Col-0, *bzip28/60*, and *ire1a/b* mutants. Seven-day-old Col-0 and mutant seedlings were treated with or without 2 mM DTT or 5 μg/mL Tm for 5 h. The expression levels of *AtPDI11* (**A**) and *AtBIP3* (**B**) were normalized to the expression level of *AtGAPC*. Values are shown as means ± SE from three independent biology repeats. Statistical significance compared with Col-0 was analyzed using one-way ANOVA. ** *p* < 0.01.

In Arabidopsis, UPR sensor AtIRE1 and bZIP transcription factors, such as AtbZIP28 and AtbZIP60, were key components of UPR signaling pathway [7]. It was reported that upregulation of *AtPDI* genes were partially modulated by AtbZIP60 [14]. To further examine whether the expression of *AtPDI11* under DTT/Tm treatment is regulated by the UPR pathway, we measured the transcript accumulation of *AtPDI11* in *ire1a/b* and *bzip28/60* mutants exposed to ER stress [33]. As a positive control, the transcript level of *AtBIP3* was significantly increased by DTT or Tm treatment in wild-type Col-0. However, the induction of *AtBIP3* was markedly decreased in *ire1a/b* and *bzip28/60* mutants compared with that in wild-type plants. Similar phenomena were observed for *AtPDI11* in *ire1a/b* and *bzip28/60* mutants compared to wild-type plants (Figure 5). This result suggests that the deletion of UPR sensor IRE1 and transcription factors bZIP28/60 almost completely prevented the transcriptional upregulation of *AtPDI11* under ER stress, and the induction of *AtPDI11* by ER stress is governed by key UPR signaling mediators.

### 2.5. AtPDI11 D Domain Is Required for Its Oxidative Protein Folding Activity

**Figure 6:**
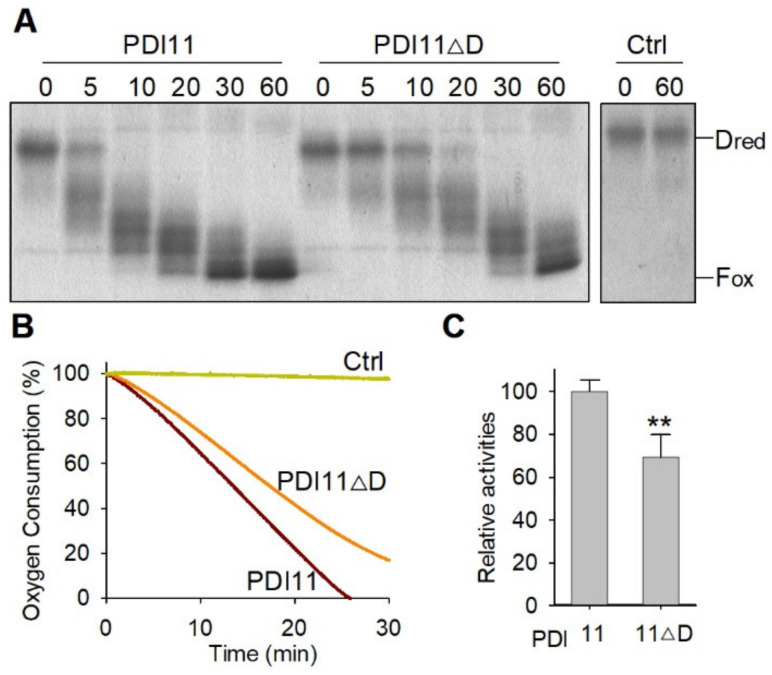
The **D** domain is required for the enzyme activities of AtPDI11. (**A**) Analysis of AtPDI11 and AtPDI11Δ**D** activities based on gel-based RNase A refolding assay. In total, 8 μM denatured and reduced RNase A was incubated in buffer B containing 3 μM AtPDI11 or AtPDI11Δ**D**, 3 μM AtERO1, and 100 μM FAD at 25 °C. The same volume of sample from the reaction mixture was quenched with AMS at various time points and separated by 15% non-reducing SDS-PAGE. Dred: denatured and reduced RNase A; Fox: fully oxidation RNase A. Control: Reaction without AtPDI11 or AtPDI11Δ**D**. (**B**) Comparison of enzyme activity between AtPDI11 and AtPDI11Δ**D** by oxygen consumption assays. Oxygen consumption was monitored in buffer B containing 2 μM AtERO1, 20μM AtPDI11 or AtPDI11Δ**D**, 20 μM FAD and 10 mM GSH, and GSH was supplied as reducing equivalents. Control: Reaction without AtPDI11 or AtPDI11Δ**D**. (**C**) Statistical analyses of enzyme activity between AtPDI11 and AtPDI11Δ**D**. Relative enzyme activities were estimated by measuring the slope of the linear phase of the oxygen consumption curve in (**B**) after subtracting the control. Values are the means of three biological repeats ± SE (*n* = 3). Statistical significance was determined by Student’s *t*-tests. ** *p* < 0.01.

AtPDI11 is the only member of the PDI family that possess a **D** domain [30]. However, the function of the **D** domain is currently unknown. In order to investigate the function of the **D** domain in oxidative protein folding, we purified the recombinant His-FLAG-AtPDI11 and His-FLAG-AtPDI11Δ**D** (a truncated PDI11 lacking **D** domain) (Supplementary Figure S2). To examine the oxidative folding activities of AtPDI11 and AtPDI11Δ**D**, we first employed an AtERO1-AtPDIs-Dred (reduced and denatured) RNase A system, where AtPDIs relay the disulfide bonds from AtERO1 to Dred RNase A in vitro. The oxidative folding of Dred RNase A protein can be evaluated by examining changes of molecular weight (MV), as blocking of free thiols of Dred RNase A by 4-acetamido-4′-maleimidylstilbene -2, 2′-disulfonic acid (AMS) results in increases in its MV by 0.5 kDa per thiol residue [34]. As shown in the Figure 6A, the full-length AtPDI11 converted most of Dred RNase A into partially oxidized intermediates (Pox) within 10 min, and converted into full oxidized (Fox) forms at 60 min. Although AtPDI11Δ**D** was also able to catalyze the conversion of Dred RNase A into Pox and Fox states, the catalytic efficiency of AtPDI11Δ**D** was much lower than that of PDI11 (Figure 6A). This was confirmed in the oxygen consumption assay (Figure 6B,C). These results indicate that lacking the **D** domain seriously affects the activity of PDI11 to oxidize the substrates in vitro.

### 2.6. The D Domain Is Required for the Role of AtPDI11 When Plants Are Grown under Reducing Conditions

**Figure 7:**
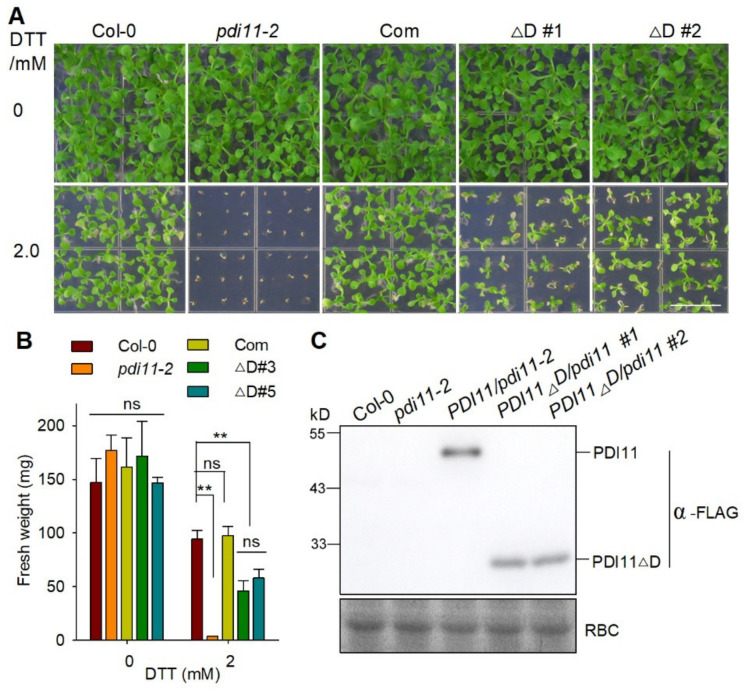
The **D** domain is required for AtPDI11′s function under reducing conditions. (**A**) The growth phenotype of *AtPDI11Δ**D*** gene expressing in *pdi11-2* mutant transgenic plant. Col-0, *pdi11-2*, Com, and *proPDI11::PDI11Δ***D** seedlings were grown on medium with or without 2.0 mM DTT for 2 weeks. Bar = 1 cm. (**B**) Statistical analyses of the fresh weight of plants presented in (**B**). Values are shown as means ± SE from three independent biology repeats (*n* = 36). Statistical significance compared to Col-0 was determined by one-way ANOVA. ** *p* < 0.01; ns, not significant. (**C**) The expression of *AtPDI11-FLAG* and *AtPDI11Δ**D**-FLAG* transgenic plants in *pdi11-2* background. The expression proteins were detected by Western blotting with an anti-FLAG antibody. Rubisco (RBC) stained with Coomassie blue shows roughly equal loading.

To further confirm the function of the **D** domain genetically, we transformed *pdi11-2* mutant plants with a construct containing *PDI11Δ**D*** driven by the native promoter of *AtPDI11*. As shown in Figure 7, there was no obvious difference between Col-0, *pdi11-2*, Com, and *AtPDI11Δ**D***/*pdi11-2* under normal growth conditions. When grown on medium containing 2 mM DTT, the Com plants displayed a similar growth phenotype to Col-0 plants. However, the expression of AtPDI11Δ**D** only partially rescued the growth inhibition of *pdi11-2* under reducing conditions. Taken together, these results suggest that the **D** domain is required for AtPDI11′s function in vivo under reducing conditions.

## 3. Discussion

In eukaryotic cells, the ERO1-PDI protein folding pathway acts as an important oxidative protein folding system. PDI-S subgroup isoform, unlike canonical PDI sharing **a**-**b**-**b′**-**a′** arrangement, with an **a**-**a′**-**D** domain arrangement and lacking **b′** domain, is a unique PDI [14,30]. It has no ortholog in animals and has been identified only in plants and *Dictyostelium discoideum* [29,30,35]. However, the expression regulation and functional mechanisms of this PDI isoform are not fully understood.

In this work, we showed that PDI-S subgroup member is widely distributed and appear to be present throughout revolution from lower plants (algae and mosses) to higher plants (gymnosperms and angiosperms) (Figure 1). In Arabidopsis, PDI-S subgroup member *AtPDI11* was upregulated under different concentrations of DTT and Tm (Figure 2, Figure 3 and Figure 4). The induction of *AtPDI11* by ER stress can be impaired in *bzip28/60* or *ire1a/b* double mutants, suggesting it is mediated by the UPR signal pathway (Figure 5). Both oxygen consumption assays and gel-based RNase A refolding assay showed that **D** domain is critical for the activities of AtPDI11 (Figure 6). These results have also been verified in the transgenic complementation lines (Figure 7). Recently, we showed that the *pdi11* single mutant, but not other PDI member single mutants, dramatically inhibited growth at seedling stage, although PDI-M family members AtPDI9/10 or PDI-L family members AtPDI2/5/6 exhibited higher activities to catalyze the Dred RNase A refolding [25]. These results suggest that, at least at the seedling stage, AtPDI11 may play an irreplaceable role in catalyzing the folding of specific substrates.

It was reported that the transcription of *AtPDI11* gene was induced by ER stress [14]. In this work, we found that the deletion of UPR sensor IRE1 and transcription factors bZIP28/60 almost completely prevented the transcriptional upregulation of AtPDI11 under ER stress. These results indicate that the induction of AtPDI11 by ER stress is governed by key UPR signalling mediators (Figure 5). AtBIP3 is a key chaperone that is induced by UPR to boost the protein-folding capacity in the ER. Notably, the expression of *AtBIP3* is significantly lower in *bzip28/60* in normal conditions in our work. This result is in line with previous reports that *AtBIP1* was lower expression level in *bzip28/60* than that in wild-type plants [36] and suggests that *AtBIP3* transcription predominately relies on bZIP28 and bZIP60. IRE1 is a transmembrane ER stress sensor. It was demonstrated in yeast and animals that BIP can bind to the luminal domain of IRE1 under normal conditions [37]. In our work, *AtBIP3* expression is higher in *ire1a/b* than that in wild type. However, according to Ruberti et al., there was no significant difference in the expression of *AtBIP3* between *ire1a/b* mutant and wild-type plants [38]. This needs to be investigated in detail in future studies.

**Figure 8:**
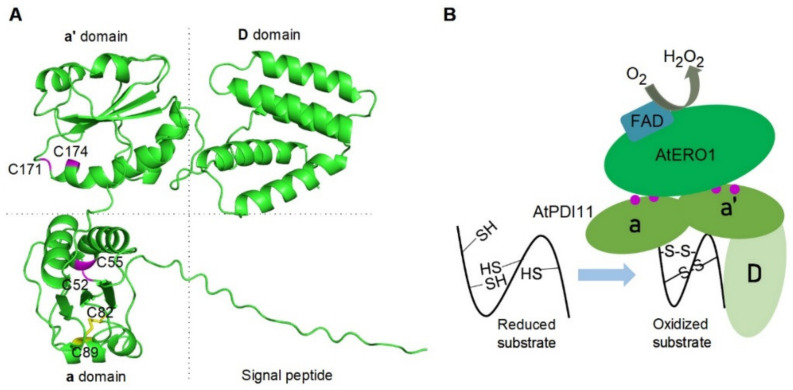
Schematic model illustrating the pathway of AtPDI11 catalyzing disulfide bond formation in the ER. (**A**) Ribbon representation of AtPDI11 generated by AlphaFold prediction (https://www.alphafold.ebi.ac.uk). AtPDI11 is composed of two thioredoxin-like domains (**a** domain and **a′** domain) and **D** domain. The active sites of **a** domains are shown in lilac and the intermolecular disulfide bond Cys82_Cys89 is indicated by a yellow region. (**B**) Schematic representation of the thiol-disulfide interchange reactions catalyzed by AtERO1 and AtPDI11. The **a**-**a′** region of AtPDI11 can interact with AtERO1. AtERO1 is oxidized by molecular oxygen to produce peroxide in the presence of FAD. AtPDI11 accepts oxidizing equivalents from AtERO1 and further oxidizes the free sulfhydryl groups of the substrate to catalyze the disulfide bond formation. Lavender solid circles indicate the active sites in **a** or **a′** domains of AtPDI11.

It has been reported that the **b′xa′** fragment of HsPDI serves as the minimal element for its binding to HsEro1α [39]. In previous studies, we found that the **a**-**a′** region, but not **D** domain of AtPDI11, provides the binding site for AtERO1 [25]. Deletion mutation analysis and genetic complementation experiment also demonstrated that the **D** domain is necessary for the ability of AtPDI11 to catalyze oxidative protein folding (Figure 6 and Figure 7). The **a**-**a′** region of PDI11 also provides a binding site to substrate because the AtPDI11 mutant lacking **D** domain is still able to catalyze Dred RNase A at a slower rate compared with the WT AtPDI11 (Figure 6). In order to further investigate the function of **D** domain, we analyzed the three-dimensional structure of AtPDI11 using AlphaFold prediction [39]. As shown in Figure 8A, it seems clear that AtPDI11 is comprised of two **a**-type domains and a C-terminal five-α-helical **D** domain, except for an N-terminal signal peptide. The three domains located at three corners form a triangle shape. In humans, the C-terminal domain of ERp29 is demonstrated to be the conserved 5-α-helix hydrophobic region that provides a protein/peptide binding site [27]. Recently, we found that AtPDI11 interacts with Arabidopsis calreticulin 1 (CRT1) and 2. Furthermore, it is the **D** domain rather than the **a**-**a′** region which provides the binding site for CRT1/2 [31]. Therefore, we hypothesize that the triangle region of AtPDI11 may also provide a hydrophobic environment to contribute to oxidative folding of substrates. Taken together, we propose the following model: AtERO1 is oxidized by molecular oxygen in an oxidative reaction which generates peroxide in the presence of FAD cofactor, AtERO1 interacts with the **a**-**a′** region of AtPDI11, and reduced substrate may bind to a hydrophobic region composed of three domains of AtPDI11. Finally, AtPDI11 accepts oxidizing equivalents from oxidized AtERO1 and further oxidizes the free sulfhydryl groups of the reduced substrate to catalyze disulfide bond formation (Figure 8B).

AtPDI11, unlike other PDI family members, does not contain a C-terminal KDEL motif that is capable of effectively limiting localization to the ER lumen [14]. Some evidence for the involvement of **D** domain in ER retention have been discovered. Monnat et al. found that, in *Dictyostelium discoideum* Dd-PDI with **a**-**a′**-**D** modular organization was localized in the ER and its 57 residue C-terminal domain is critical for ER retention of Dd-PDI, meanwhile, green fluorescent protein (GFP) fused in frame with 57 residue C-terminal amino acids of Dd-PDI was also localized to the ER [35]. In contrast, in both transient expression and stable transgenic plants, there was no obvious difference between the full length and a truncation of AtPDI11 lacking **D** domain in the localization of the ER [28]. In our work, AtPDI11Δ**D** driven by its native promoter largely rescued the growth inhibition of the *pdi11* mutant under reducing conditions (Figure 6). These results indicate that the **D** domain, at least in Arabidopsis, cannot be the sole determinant of ER retention in the PDI-S subgroup. 

## 4. Materials and Methods

### 4.1. Plant Materials and Growth Conditions

The plants *pdi11-1* (SALK_135268C), *pdi11-2* (SALK_148421), and *proPDI111::PDI11-FLAG/pdi11-1* were reported previously [30]. *ire1a/b* was generated by crossing *ire1a-2* and *ire1b-4* single mutant [33]. All plants were grown on soil in a growth chamber with a 16-h light/8-h dark lighting condition at 22 °C. For seedlings grown on medium, seeds were surface sterilized and sown in germination medium (1% (*w*/*v*) sucrose, 0.8% (*w*/*v*) agar, and 2.5 mM MES at pH 5.7) with or without different concentration of DTT or Tm. Before moving to the growth chamber, the plates were first kept at 4 °C for 2 days to break dormancy. 

### 4.2. Plasmid Construction and Generation of Transgenic Plants

For the expression of recombinant proteins in *E.coli*, *His-FLAG-AtPDI11*, *His-FLAG-AtPDI11Δ**D*** (a AtPDI11 truncation lacking the D domain, 1–250 amino acids), and *GST-AtERO1* were generated as described previously [34]. *proPDI11::PDI11-FLAG* was generated as described previously, and *proPDI11::PDI11Δ**D**-FLAG* was generated using the same method and vector. For GUS histochemical assay, 2.0 kb upstream of the start codon for AtPDI11 was cloned into the pCAMBIA1391 binary plasmid vector fused with the β-glucuronidase (*GUS*) reporter gene at the BamHI and HindIII sites.

### 4.3. Recombinant Proteins Expression and Purification

The recombinant AtPDI11 and AtPDI11Δ**D** proteins were expressed in *E. coli* BL21 (DE3) cells by the addition of 0.5 mM isopropyl thiogalactoside at 25 °C for 10 h and purified using Ni-NTA agarose (Qiagen, Hilden, Germany) and following the manufacturer’s instruction. The recombinant GST-AtERO1 proteins were expressed in *E. coli* 21 (DE3) cells and purified as previously described [40]. Then GST tag moiety was removed by digesting GST-ERO1 proteins with PreScission Protease (Amersham Pharmacia Biosciences, Piscataway, NJ, USA). The concentration of each recombinant proteins was determined, and aliquots were stored in buffer A (50 mM Tris-HCl and 150 mM NaCl, pH 7.6) at −80 °C [8,26].

### 4.4. Phylogenetic Analysis and Sequence Alignment

Phylogenetic Analysis and Sequence Alignment was performed as described previously [32].

### 4.5. RNA Extraction and Real-Time Quantitative Polymerase Chain Reaction (RT-PCR)

Seven-day-old seedlings were treated with 2 mM DTT or 5 μg/mL Tm for 5 h. Total RNA of seedlings was isolated using TransZol (Transgen, Beijing, China) following the manufacturer’s instructions. In total, 1 μg DNase-treated total RNA was used for synthesis of first-strand cDNA in 20 μL reactions. qRT-PCR was performed on Bio-Rad CFX96 Real-Time PCR system using a PerfectStart^®^ Green qPCR SuperMix (Transgen, Beijing, China). *AtGAPC* was used for internal reference genes [41]. The primers used are listed in Supplementary Table S1.

### 4.6. Histochemical Staining

Histochemical staining for GUS activity was performed as described previously [42]. Plants grown on medium were harvested and immediately submerged in a solution (100 mM sodium phosphate (pH 7.0), 0.5 mM potassium ferrocyanide, 0.5 mM potassium ferricyanide, 1 mg/mL 5-bromo-4-chrolo-3-indolyl-β-d-glucuronide (Sigma-Aldrich, Burlington, MA, USA), and 0.1% (*v*/*v*) Triton X-100) at 37 °C in the dark for 1 h (DTT/Tm-induced GUS expression analysis). Chlorophyll was removed by submerging samples in anhydrous ethanol two times and in 70% (*v*/*v*) ethanol three times. The samples were photographed using a Nikon AZ100 5X microscope.

### 4.7. Oxygen Consumption Assay

The oxygen consumption assay was performed as described previously [39]. Oxygen consumption was monitored with an Oxygraph Clark-type oxygen electrode (Hansatech Instruments, Kings Lynn, UK). All experiments were performed in buffer B (100 mM Tris-HAc, 50 mM NaCl, and 1 mM EDTA, pH 8). All components, except for AtERO1, of each reaction were freshly mixed in a total volume of 0.5 mL, and the reaction was initiated by the injection of AtERO1 into the reaction vessel of the oxygen electrode.

### 4.8. Gel-Based Denatured and Reduced RNase A Reoxidation Analyses

Gel-based RNase A reoxidation analyses were performed as described previously [39]. In total, 3 μM AtPDI11 or AtPDI11Δ**D**, 3 μM AtERO1, 100 μM FAD, and 8 μM denatured and reduced RNase A were freshly mixed in buffer B. At various time points, the same volume of sample from the reaction mixture was quenched in addition of 5 x SDS loading buffer with 10 mM AMS (Invitrogen, Carlsbad, CA, USA) and separated by 15% non-reducing SDS-PAGE. The proteins were stained with Coomassie Blue.

## Data Availability

The data supporting the findings of this study are available within the article and its Supplementary Materials.

## Supplementary Materials

The following supporting information can be downloaded at: https://www.mdpi.com/article/10.3390/ijms23031409/s1.
