# Supplementary material for: Expression Characterization of AtPDI11 and Functional Analysis of AtPDI11 D Domain in Oxidative Protein Folding
Source: Int J Mol Sci. 2022 Jan 26;23(3):1409. doi: 10.3390/ijms23031409 (PMC8836223; doi:10.3390/ijms23031409)
Supplement: Supplementary file 1 [file ijms-23-01409-s001.zip › ijms-1548053-supplementary-done.pdf]

International Journal of
Molecular Sciences

MDPI

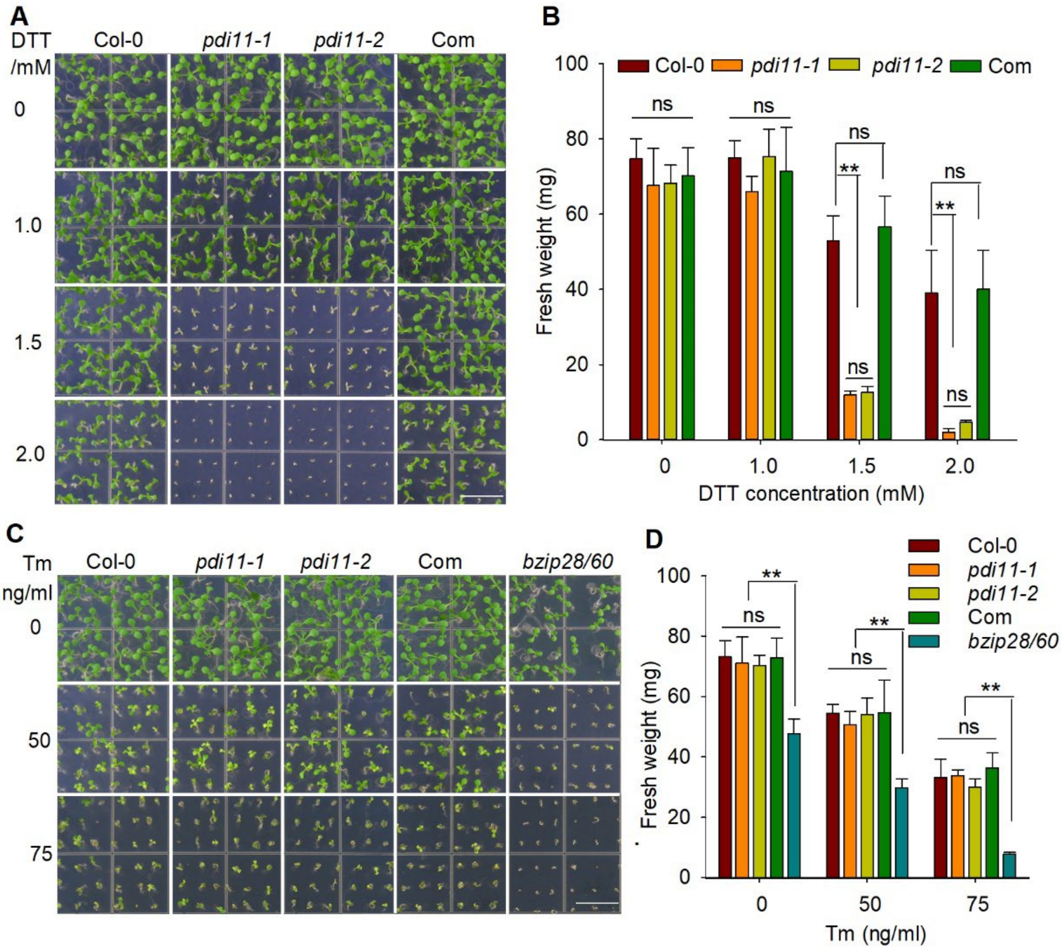

Figure S1. The growth phenotypes of *AtPDI11* T-DNA insertion mutants under different concentration of DTT/Tm treatment. (A) The growth of Col-0, *pdi11-1*, *pdi11-2* and *pdi11-1* expressing *AtPDI11* driven by its native promoter (Com) under different concentration of DTT (0, 1.0, 1.5 and 2.0 mM). Bar = 1 cm. (B) Statistic analyses of the fresh weight of plants presented in (A). Col-0, *pdi11-1*, *pdi11-2* and Com seedlings were grown on medium with or without different DTT concentration for 7 days. Values are shown as means ±SE from three independent biology repeats ($n = 36$). Statistical compared to Col-0 significance was determined by one-way ANOVA: $^{**}p < 0.01$; ns, no significant. (C) The growth of Col-0, *pdi11-1*, *pdi11-2*, Com and *bzip28/60* double mutants under different concentration of Tm (0, 50 and 75 ng/ml), and the phenotype of *bzip28/60* under Tm treatment was shown as the positive control. Bar = 1 cm. (D) Statistic analyses of the fresh weight of plants presented in (C). Col-0, *pdi11-1*, *pdi11-2* and Com seedlings were grown on medium with or without different Tm concentration for 7 days. Values are shown as means ±SE from three independent biology repeats ($n = 36$). Statistical compared to Col-0 significance was determined by one-way ANOVA: $^{**}p < 0.01$; ns, no significant.

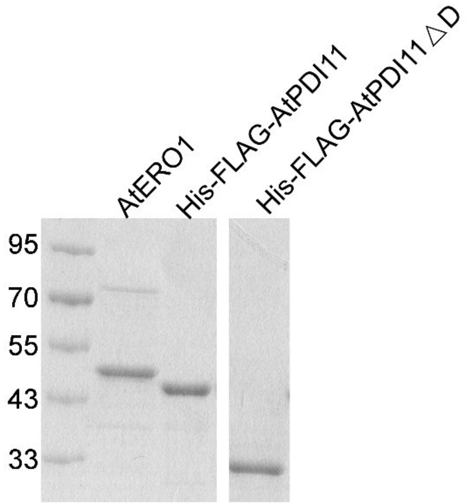

Figure S2 Expression and purification of recombinant His-FLAG-AtPDI11, His-FLAG-AtPDI11Δ**D** and AtERO1 fusion proteins.

Table S1: Primers used in this study.

Name	Sequence (5’→3’)
QRT-AtPDI11-F	GGCTAAGTCTGTGTTGATTGC
QRT-AtPDI11-R	ATTGCGTGGACCCTCATA
QRT-AtBIP3-F	CACGGTTCCAGCGTATTTCAAT
QRT-AtBIP3-R	ATAAGCTATGGCAGCACCCGTT
QRT-AtGAPC-F	TTGGTGACAACAGGTCAAGCA
QRT-AtGAPC-R	AAACTTGTCGCTCAATGCAAT
AtPDI11-BamHI-F	CGGGATCCATGGCGAAATCTCAGATCTG
AtPDI11-StuI-R	GAAGGCCTAGAAGAAGCAACGAACGTGG
AtPDI11**ΔD**-StuI-R	GAAGGCCTCCCAGATTTCTCGTTGATG
proPDI11-HindIII-F	CCCAAGCTTGTACGAAATGTAGGTG
proPDI11-BamHI-R	CGGGATCCTTTTTTTTCTGCTTCTCAC
